# Protective effects of PACAP in ischemia

**DOI:** 10.1186/s10194-018-0845-3

**Published:** 2018-03-02

**Authors:** Dora Reglodi, Alexandra Vaczy, Eloísa Rubio-Beltran, Antoinette MaassenVanDenBrink

**Affiliations:** 1 Department of Anatomy, MTA-PTE PACAP Research Group, University of Pecs Medical School, Pécs, Hungary; 2 Department of Internal Medicine, Division of Vascular Medicine and Pharmacology, Erasmus MC, Rotterdam, The Netherlands

**Keywords:** Migraine, PACAP, Ischemia, Neuroprotection

## Abstract

Pituitary adenylate cyclase activating polypeptide (PACAP) is an ubiquitous peptide involved, among others, in neurodevelopment, neuromodulation, neuroprotection, neurogenic inflammation and nociception. Presence of PACAP and its specific receptor, PAC1, in the trigeminocervical complex, changes of PACAP levels in migraine patients and the migraine-inducing effect of PACAP injection strongly support the involvement of PACAP/PAC1 receptor in migraine pathogenesis. While antagonizing PAC1 receptor is a promising therapeutic target in migraine, the diverse array of PACAP’s functions, including protection in ischemic events, requires that the cost-benefit of such an intervention is well investigated by taking all the beneficial effects of PACAP into account. In the present review we summarize the protective effects of PACAP in ischemia, especially in neuronal ischemic injuries, and discuss possible points to consider when developing strategies in migraine therapy interfering with the PACAP/PAC1 receptor system.

## Introduction

PACAP is an ubiquitous peptide discovered almost three decades ago [1], and it has been described to be involved in neurodevelopment, neuromodulation, neuroprotection, neurogenic inflammation and nociception [2]. It belongs to the vasoactive intestinal peptide (VIP)/glucagon/growth hormone releasing factor/secretin superfamily [2] and is encoded by the ADCYAP1 gene, located on chromosome 18, which expresses a proprotein that is further processed into multiple mature peptides. Alternative splicing results in multiple transcript variants, including two forms that contain either 27 or 38 amino acids (PACAP27 and PACAP38). Since in mammals PACAP38 is the most prevalent form [3], in this review PACAP38 will be referred to simply as PACAP unless stated otherwise.

PACAP exerts its functions through the activation of three different G-protein coupled receptors (GPCRs): VPAC1, VPAC2 and PAC1. While VPAC1 and VPAC2 receptors are coupled to Gs proteins and show similar affinity for VIP, the PAC1 receptor has a 100-fold selectivity for PACAP27 and PACAP38 over VIP, leading to the activation of adenylate-cyclase and phospholipase C signaling transduction pathways [4].

In the central nervous system (CNS), PACAP has been described in the pituitary, thalamus, hypothalamus, hippocampus, locus coeruleus, periaqueductal grey area, the dorsal horn of the spinal cord and in astrocytes [5–14]. Of special interest, PACAP is expressed in the trigeminal nucleus caudalis (TNC) and trigeminal ganglia [15], which could suggest a possible role for PACAP in migraine pathogenesis. In rats, injection of PACAP into the paraventricular nucleus of the hypothalamus increases the activity of the TNC, which can be reverted by administration of the PAC1 receptor antagonist [16], and intrathecal injection of PACAP induces hyperalgesia [8]. PACAP plasma levels in migraineurs are elevated during a migraine attack, in comparison with the interictal levels [17]. Most importantly, if injected peripherally to migraineurs, PACAP is able to induce an immediate headache in 90% of the cases, that is followed by a delayed migraine-like headache in almost 60% of the subjects; conversely, only 15% of the healthy controls experience the delayed migraine-like headache [15]. These findings are similar to those obtained after peripheral administration of calcitonin gene-related peptide (CGRP) [18]. Interestingly, PACAP is a weaker dilator of the human meningeal artery when compared to VIP [19]. Since VIP was earlier reported not to induce migraine-like headaches [20], this could suggest that the role of PACAP in migraine is probably through modulation of the trigeminocervical complex via the PAC1 receptor.

In view of the suggested role of PACAP, but not VIP, in migraine, an antibody against the PAC1 receptor (AMG 301) has been developed for the treatment of migraine (Clinical trials identifier: NCT03238781). In preclinical studies, AMG 301 has been shown to inhibit stimulus-evoked nociceptive activity in the TNC and the results are comparable to the inhibition observed with sumatriptan, supporting the role of PAC1 receptor in migraine pathophysiology. However, it is important to consider the ubiquitous nature of PACAP and its receptors, since they have also been described to be widely expressed in the periphery, such as in the thyroid and parathyroid glands, lungs, pancreas, liver, colon, stomach and blood vessels [3, 11, 21–25]; thus they participate in several respiratory, gastrointestinal, reproductive and cardiovascular (patho) physiological processes [2] and, as it will be discussed, play a significant role in the homeostatic responses to ischemic events [26–32], Table 1.

**Table 1:** Summary of the protective effects of PACAP in different ischemic models, human diseases and changes of PACAP levels and PAC1 receptor expression in ischemic conditions

Ischemic models		Lesion size	Degree of functional deficit	PACAP level	PAC1 receptor expression
Cerebral ischemia	Global ischemia in mouse and rat: 4VO, BCCAO	↓ [48, 72]	NA	↓ PACAP 38 [64]- in CA1, [68]-in hippocampus granule cells ↑PACAP 38 [72]- in hippocampus	↓ [68, 70]- in hippocampus, [69]- in hippocampal astrocytes
	Focal ischemia in mouse and rat: MCAO	↓ [27, 36, 40–47, 58, 67, 71]	↓ [27, 46, 47]	↑ PACAP 38 [56]- in brain, [67]- in cortical pyramidal cells ↓ PACAP 38 [58]- in cortex, striatum, subcortical area	↑[65]- in cortex, caudate, putamen, [67]- in neurons and astrocytes
	MCAO+BCCAO in rat	↓ [55]	↓ [55]	↑ PACAP 38 [55]- in cortex	N/A
	Stroke, hemorrhages in human	N/A	N/A	↑ PACAP 38 [55]- penumbral region, [69]- intracerebral, [70]- subarachnoid]	N/A
Retinal ischemia	Transient ischemia: high intraocular pressure	↓ [83]	N/A	N/A	N/A
	Permanent ischemia: BCCAO	↓ [76, 81, 82, 87, 88, 90]	↓ [81]	N/A	N/A
Cardiac ischemia	Ischemia reperfusion in rat and human	↓ [94, 95]	N/A	↓ PACAP 38 [91]- in stellate ganglion ↑ PACAP 38 [91]- in heart ↑ PACAP38-Li, PACAP 27-Li [92]- in heart	↑ [92]
Liver ischemia	Ischemia reperfusion in mouse	↓ [96]	N/A	↑ PACAP 27/38 [96]	↑ [96]
Intestinal ischemia	Ischemia reperfusion in mouse and rat	↓ [97, 98]	N/A	↓ PACAP 38 [97]	N/A
Kidney ischemia	Ischemia reperfusion in mouse, rat, and in human	↓ [100–104]	N/A	N/A	N/A

*Abbreviations*: *MCAO* middle cerebral artery occlusion, *4VO* 4 vessel occlusion, *BCCAO* bilateral common carotid artery occlusion, *-Li* -like immunoreactivity, *N/A* not applicable, not measured

## Review

### PACAP in brain ischemia

PACAP has been shown to be neuroprotective in vitro in different neuronal cultures against various toxic insults and in models of neuronal injuries in vivo [33, 34]. Numerous in vivo data have been published showing its protective actions in cerebral ischemia [33, 35]. The first proof for the in vivo neuroprotective effect came from a rat global ischemia study, where intravenous or intracerebroventricular (icv) PACAP administration reduced hippocampal neuronal loss [36]. This was achieved via suppression of JNK and p38, while stimulation of ERK activity [37–39]. These observations were followed by studies demonstrating that PACAP was also effective in transient and permanent focal ischemia in rats and mice induced by middle cerebral artery occlusion (MCAO) [27, 40–44].

Subsequent studies provided further details on the neuroprotective mechanisms. Anti-apoptotic and anti-inflammatory actions seem to be the main protective mechanisms in PACAP’s actions in rat and mouse models of cerebral ischemia. PACAP decreased apoptosis in the ischemic penumbra [45], inhibited expression of bcl-2-associated death promoter, caspase-3, macrophage inflammatory protein-1alpha, inducible nitric oxide synthase2, tumor necrosis factor-(TNF) alpha mRNAs and increased ERK2, bcl-2 and IL-6 [40, 41, 46]. Decreased inflammatory response was also found after post-stroke PACAP-producing stem cell transplantation, where numerous chemokines as well as TNF, NFkappaB and IL-1 decreased [47]. In brain cortical neurons subjected to oxygen-glucose deprivation and reoxygenation, PACAP induced neuronal protection by both direct actions through PAC1 receptor, and indirect pathways via neurotrophin release, activation of trkB receptors and attenuation of neuronal growth inhibitory signaling molecules p75NTR and Nogo receptor [41]. In addition, PACAP induced apurinic/apyrimidinic endonuclease APE1 in hippocampal neurons that can be an additional factor reducing DNA stress and hippocampal CA1 neuronal death in global ischemia [48]. In mouse MCAO, several genes were affected in the ischemic core and penumbra after PACAP treatment [49–52]. Among the upregulated genes was IL-6, which was strongly induced during the critical first 24 h, suggesting a relationship between PACAP and IL-6 in accordance with previous findings by Ohtaki and co-workers [40]. Several other cytokines and growth factors were altered in a region-specific and time-dependent fashion after post-ischemic PACAP treatment, such as brain derived neurotrophic factor [50, 51]. Whether alterations of these factors are consequences of PACAP reducing infarct volume by other mechanisms or represent a causative factor is not known at the moment. Only in case of IL-6, it has been proven that PACAP failed to improve ischemic lesion in IL-6-deficient mice, showing the causative role of IL-6 in PACAP-mediated neuroprotection in mice [40]. Numerous further factors playing a role in neuronal defense, axonal growth and development were also modified after ischemia [52]. A relationship between hypoxia inducible factor (HIF) and PACAP was described in several studies in different experimental paradigms [53–55]. Under in vitro and in vivo hypoxic conditions, HIF1-alpha activation upregulated PACAP, which in turn activated PAC1 receptor [56]. Although PACAP reduced HIF1-alpha expression in a model of diabetic retinopathy 2 weeks after the treatment, bone marrow-derived stem cells homing into the ischemic brain was also facilitated by a recently described HIF1-alpha-activated PACAP38-PAC1 signaling process [55]. A detailed time-dependent analysis of PACAP’s effect on cerebral HIF1 expression could clarify the role of this pathway in PACAP-induced neuroprotection in ischemia. Analogs of PACAP were also tested in focal ischemic models. In a study of ischemia/reperfusion injury, a potent metabolically stable PACAP38 analog [acetyl-(Ala^15^, Ala^20^) PACAP38-propylamide] led to the same degree of protection as native PACAP38 [46]. This is an important finding, as one of the limitations of PACAP’s therapeutic use is its poor stability. However, according to these data enhancing its plasmatic half-life did not lead to an increase of its neuroprotective potential [46], but analogs might have less vasomotor side effects, as described in another study [57].

As far as functional recovery is concerned, PACAP is able to improve functional deficits in association with the morphological amelioration in stroke models. In rat permanent focal cerebral ischemia, PACAP improved certain sensorimotor deficits, such as reaction times to body surface touch [27]. Another study further supported this in a transient MCAO, evaluating neurological impairment by degree of limb flexion, grasping and symmetry of movements [46]. In a permanent focal ischemia model, PACAP-producing stem cells transplanted icv 3 days after stroke promoted functional recovery even when given beyond the therapeutic window for structural recovery [47].

PACAP is known to cross the blood-brain barrier (BBB), but it is still questionable whether the rate is sufficient to lead to effects in the brain under physiological or pathological conditions [2, 38]. Although ischemic conditions change region-specific crossing, it is suggested that the passage is sufficient enough to induce neuroprotection in ischemic brains [58]. Antisenses inhibit efflux pumps of the BBB, and could inhibit PACAP27 efflux and reduce the infarct size in mouse focal ischemia [59]. Regarding changes in cerebral blood flow, in some studies PACAP increased cerebral blood flow in ischemic conditions, while in others no change or even decrease was found [27, 46, 60]. PACAP has potent vasodilatory effects, which can also be included in the pathomechanism of migraine [61–63]. However, given the contradictory data on cerebral blood flow after PACAP treatment, it remains unknown at the moment whether this effect plays a role in post-ischemic neuroprotection.

The role of endogenous PACAP was suggested by upregulation of PACAP signaling in different ischemia models and from knockout studies (Table 1). In a gerbil model of global ischemia, decrease in PACAP expression was followed by an increase 5 days later. This was accompanied by increases in PAC1 receptor expression in the vulnerable CA1 region, in contrast to the more resistant CA3 area, where PACAP expression did not change [36, 64]. Upregulation of PAC1 receptor could also be observed after focal ischemia [65, 66]. A massive upregulation of PACAP was found in peri-infarct regions [67]. In a rat global ischemia model, moderate PAC1 mRNA decrease was observed throughout the hippocampus, while granule cells showed increased PACAP expression [68]. It was suggested that the altered PACAP and PAC1 receptor expression might play a role in regulated neurogenesis after stroke [68]. In mouse hippocampal astrocytes, PAC1 receptor expression was increased 7 days after stroke, suggesting an important role of PACAP in reactive astrocytes [69, 70]. Further evidence for the endogenous protection by PACAP came from studies using PACAP deficient mice. Hetero- and homozygous PACAP knockout animals had increased infarct volume with increased edema formation and more severe neurological deficits after MCAO, and these could be ameliorated by PACAP injection [40, 71]. Furthermore, cytochrome-*c* release was higher, while mitochondrial bcl-2 was lower in mice lacking PACAP. It was also suggested that these protective effects could be mediated in part by IL-6 [40]. Endogenous PACAP also promotes hippocampal neurogenesis after stroke, as proliferation of neuronal stem cells in the subgranular zone of the hippocampus was found to be increased in wild type mice, but not in PACAP heterozygous animals [72].

The few available human data also support that PACAP might play a role in ischemic neuronal conditions. It was hypothesized that the elevated blood PACAP levels may reflect an increased leakage into circulation or an overproduction of PACAP as a pathological response to the loss of neural tissue in the CNS and it might be associated with the neuroprotective effects of the neuropeptide [73]. Plasma PACAP concentrations were higher in patients after acute spontaneous basal ganglia and aneurysmal subarachnoid hemorrhages than in healthy control subjects [73, 74]. Positive association was shown between PACAP levels and neurological score, as well as with hematoma volume. Patients, who died within the first week after admission, had higher PACAP levels and overall survival times were shorter in individuals with high PACAP concentrations [73, 74]. It is suggested that PACAP could be a good prognostic predictor in hemorrhage patients. These studies suggest that PACAP can be an independent predictor of survival and a potential prognostic biomarker of brain hemorrhage.

## PACAP in retinal ischemia

PACAP is considered to be a potent neuroprotective peptide with potential therapeutic use also in retinal diseases [34, 75–79]. Similarly to models of cerebral ischemia, protective effects have been described in animal models of retinal ischemia. Intravitreal injection of PACAP38 or PACAP27 following bilateral common carotid artery occlusion in rats preserved the thickness of all retinal layers and reduced cell loss in the ganglionic layer. Immunohistochemistry demonstrated that PACAP rescued fully or partially several retinal cell types from ischemia-induced damage. The PACAP antagonist PACAP6–38 could block these protective effects [76, 80]. Electroretinography showed that ischemia caused functional loss in the retina, whereas PACAP treatment resulted a preserved retinal function [81]. Endogenous PACAP had similar protective effects, as knockout mice were more susceptible to retinal ischemic injury [82]. Efficacy of PACAP was also shown in another retinal ischemia model induced by high intraocular pressure, which could be blocked by a cAMP antagonist [83]. Testing possible therapeutic effects of various PACAP fragments and analogues, and three related peptides (VIP, secretin, glucagon) revealed that the most effective forms were PACAP38 and PACAP27, while the other fragments had either no effects or slight antagonistic effects [84, 85]. Related peptides had no effect except for VIP, which was retinoprotective at concentrations ten times higher than it is required for PACAP [84, 86]. Recent results have shown that PACAP38 and 27 are able to cross the ocular barriers and exert retinoprotective effects in ischemia even when given in form of eye drops [87, 88], providing the basis for an easy route of future therapeutic use.

Examining the protective mechanisms in retinal hypoperfusion, several studies have revealed possible signaling pathways resulting in neuroprotection. Another study investigated possible receptorial mechanisms. All three PACAP receptors (PAC1, VPAC1, VPAC2) are expressed in the retina, with PAC1 receptor showing dominant role in the retinoprotective effects [34]. Our research group confirmed the involvement of PAC1 receptors in the PACAP-induced retinoprotection using a selective PAC1 receptor agonist maxadilan in permanent common carotid artery ligation. Maxadilan rescued retinal layers from ischemia-induced degeneration and decreased expression of cytokines such as CINC-1, IL-1α, and L-selectin [89]. In another study, intravitreal PACAP increased the activation of the protective Akt and ERK1/2, while decreased both p38MAPK and JNK activation in hypoperfused retinas. After ischemia several cytokines were overexpressed (CINC, CNTF, fractalkine, sICAM, IL-1, LIX, Selectin, MIP-1, RANTES and TIMP-1), but attenuated by PACAP38 [90]. Moreover, the neuropeptide further increased vascular endothelial growth factor and thymus chemokine levels. These results suggest that PACAP can ameliorate hypoperfusion injury involving Akt, MAPK pathways and anti-inflammatory actions.

## PACAP in cardiac and other peripheral ischemic conditions

The cytoprotective effects of PACAP in ischemic conditions have also been observed outside the nervous system in various peripheral organs. Expression of PACAP mRNA increased after myocardial infarction in mice, and immunohistochemistry revealed a gp130-dependent elevation in PACAP38 in the stellate ganglion [91]. PACAP38 immunoreactivity was not detected in sham hearts, but was high in the infarct 3 days after infarction, suggesting an important role in cardiac and neuronal remodeling after ischemia-reperfusion [91]. Human data also propose the involvement of PACAP in cardiac ischemia: PACAP38- and PACAP27-like immunoreactivity was higher in ischemic heart diseases than in valve disorders [92]. Differences were also observed between ischemic and non-ischemic heart failure patient plasma, suggesting that PACAP might play an important role in the pathomechanism and progression of ischemic heart failure and it might be a potential biomarker of cardiac diseases [93]. A few available reports showed that PACAP was protective in cardiomyocyte ischemia in vitro [94, 95]. Cultured cardiomyocytes, exposed to ischemia/reperfusion, reacted to PACAP with increased cell viability and decreased apoptosis. PACAP induced the phosphorylation of Akt and protein kinaseA, while inactivated Bad, a pro-apoptotic member of the Bcl-2 family. Furthermore, PACAP increased the levels of Bcl-xL and 14–3-3 proteins, both of which promote cell survival, and decreased the apoptosis executor caspase-3 cleavage [94]. In another study, cardiomyocytes were exposed to brief preconditioning ischemia followed by 2 h ischemia and 4 h reperfusion. PACAP treatment could again increase cell viability and decrease cell death, and further reduced the level of cleaved caspase-8 under in preconditioning [95].

Numerous studies have provided evidence for the protective effects of PACAP in several other peripheral organs, like small intestine, kidney and liver. Liver ischemia/reperfusion injury triggered the expression of intrinsic PACAP and its receptors, whereas the hepatocellular damage was exacerbated in PACAP deficient mice [96]. Both PACAP27 and PACAP38 protected against hepatic ischemia, accompanied by decreased serum alanine aminotransferase levels, more preserved hepatic morphology with less cell death signs and reduced inflammation [96]. In small intestinal ischemia/reperfusion injury PACAP was protective both exogenously and endogenously. PACAP deficient mice reacted with more severe tissue damage than wild types [97, 98]. Preservation of morphological structure of small intestine after ligation of mesenteric artery followed by reperfusion was accompanied by decreased oxidative stress and increased anti-oxidant capacity in PACAP-treated animals [97, 98]. Similar results have been obtained in the kidney [99]. Both homo- and heterozygous PACAP knockout mice showed increased injury after renal artery clamping [100, 101]. Cell cultures isolated from wild type and PACAP deficient mice showed that cells from PACAP deficient mice had higher vulnerability to in vitro hypoxia [102]. In vivo, knockout mice also displayed increased tissue damage accompanied by increased inflammatory cytokine expression, decreased anti-oxidant capacity and increased expression of apoptotic markers [100, 101]. When PACAP was given as an exogenous treatment in rat renal ischemia/reperfusion injury, PACAP-treated animals had decreased mortality and inflammatory status, better preserved morphological structure in all tested histological parameters and decreased apoptotic and cytokine activity [103, 104]. All these results show that PACAP has protective effects in ischemic injuries not only in the nervous system, but also in several peripheral organs suggesting a general anti-ischemic protective role of this neuropeptide.

## Discussion

As discussed above, several in vitro and in vivo studies have shown that PACAP has protective effects in the CNS, as well as in peripheral organs during ischemic injuries [26, 31, 33, 34, 40, 41, 43, 45, 46, 89, 91, 95, 97, 101]. These actions are thought to be mediated via anti-apoptotic and anti-inflammatory mechanisms through direct activation of PAC1 receptors and indirect pathways [34, 41, 89]. Therefore, PACAP and the PAC1 receptor seem to be a promising therapeutic target for ischemic conditions [46], as well as for several neurodegenerative disorders [28, 30, 33].

Conversely, studies have shown expression of PACAP and PAC1 receptor in the TNC [15] and elevated PACAP plasma levels during migraine attacks [17]. Furthermore, the peripheral injection of PACAP induces migraine-like headaches to migraineurs [15]. This has led to the development of AMG 301, an antibody against the PAC1 receptor for the treatment of migraine, that is currently in Phase II (Clinical trials identifier: NCT03238781). As mentioned above, the PAC1 receptor was proposed as the most relevant PACAP receptor in migraine partly because this is stimulated exclusively by PACAP and not, as the VPAC1 and VPAC2 receptor, also by VIP. This latter peptide failed to induce migraine-like attacks in migraine patients [20]. Notwithstanding the evidence supporting a role for the PAC1 receptor in migraine, it cannot be completely ruled out that the differences in migraine-generation properties of PACAP and VIP are rather due to their pharmacokinetic characteristics (difference in half-life) than due to differences in their pharmacodynamic action. Thus, we feel that it is too early to exclude VPAC1 and VPAC2 as additional potential antimigraine targets.

Certainly the prophylactic treatment of migraine with AMG 301 seems promising; however, it is important to have in mind that migraineurs present an increased risk of ischemic stroke [105–109] and that PACAP and PAC1 play a key role in the homeostatic responses to ischemic conditions. Therefore, the question remains whether a mild ischemic event could transform into a full-blown infarct when PACAP’s actions are blocked; similar concerns have been raised with the novel CGRP (receptor)-antibodies [109, 110]. Although the benefits of blocking CGRP seem greater than the drawbacks, more research is warranted. Similarly, concerning blockade of the PAC1 receptor, further studies are required to determine the possible side effects of long-term blockade of PAC1 signaling, and to study whether the activation of indirect pathways involved in the protective actions of PACAP is sufficient during ischemic events [111].

Further, it remains to be established whether the same patients that show a positive therapeutic response to CGRP (receptor)-antibodies will have a positive response to PAC1 antibodies, or that both types of medications are most effective in a separate population of migraine patients, depending on the peptide that is most predominant in their individual migraine pathophysiology. In view of the role of both CGRP and PACAP in preserving homeostasis under ischemic conditions, it remains of particular interest whether these antimigraine drugs could be combined, or whether simultaneous use would augment their side-effect potential.

## Conclusions

In conclusion, PAC1 antibodies may present a valuable new tool in the treatment of migraine. Larger clinical studies will shed more light on the efficacy of these antibodies in migraine. The cardiovascular safety should be investigated in both preclinical models as well as in relevant patient populations.

## Abbreviations

BBB: Blood-brain barrier
BCCAO: Bilateral common carotid artery occlusion
CGRP: Calcitonin gene-related peptide
CNS: Central nervous system
GPCRs: G-protein coupled receptors
HIF: Hypoxia inducible factor
icv: Intracerebroventricular
IL-6: Interleukin-6
MCAO: Middle cerebral artery occlusion
PACAP: Pituitary adenylate cyclase activating polypeptide
TNC: Trigeminal nucleus caudalis
TNF: Tumor necrosis factor
VIP: Vasoactive intestinal peptide
4VO: 4 vessel occlusion
